# Age-related effects of repeated task switching in a novel voluntary gait adaptability task

**DOI:** 10.1007/s00221-023-06623-8

**Published:** 2023-04-26

**Authors:** Kyungwan Kim, Lena Deller, Marie Vinent, Wiebren Zijlstra

**Affiliations:** Institute of Movement and Sport Gerontology, German Sport University, Cologne, Germany

**Keywords:** Aging, Gait, Motor learning, Repeated task switching, Dynamic balance control, Voluntary gait adaptability

## Abstract

Age-related effects of task switching have been extensively studied based on cognitive tasks and simple motor tasks, but less on complex cognitive-motor tasks involving dynamic balance control while walking. The latter tasks may especially be difficult and relevant for older adults in terms of safe mobility in daily life. The aim of the present study was, therefore, to examine age-related changes in task-switching adaptability using a novel voluntary gait adaptability test protocol. Fifteen healthy young (27.5 ± 2.9 years) and 16 healthy old (70.9 ± 7.6 years) adults carried out 2 different visual target stepping tasks (either target avoidance or stepping) twice in a block (A–B–A–B, 2 min per task; three blocks in total) without any intrablock breaks. Our results showed that old adults showed significantly more step errors both in Tasks A and B as well as more interference effects than young adults. Age-related differences in step accuracy were significant in the anterior–posterior direction both in Task A and B but not in the mediolateral direction. Both in step errors and accuracy, no interaction effects of age and trial were shown. Our results suggest that old adults could not cope with rapid and direct task changes in our voluntary gait adaptability task as young adults. Since the significant main effect of trial for Task B, but not Task A appears to be due to different task complexity, further studies may determine the effect of task complexity or task switch timing.

## Theoretical background

While walking in daily life, old adults (OA) are often faced with various external circumstances such as slippery floors, pedestrians on a narrow street, or obstacles, which can lead to falls. The ability to voluntarily adapt walking to changing external conditions is crucial for safe mobility. Voluntary gait adaptability (VGA) requires multidimensional cognitive and motor skills and is based on continuous interaction between sensory, cognitive, and motor processes to safely maintain goal-oriented locomotion (Caetano et al. 2017). Previous studies have well investigated diverse aspects of VGA based on stepping targets, avoiding obstacles, or stepping up and down stairs while walking among OA (Yamada et al. 2011; Caetano et al. 2017) and patients with Parkinson’s disease (Mollaei et al. 2017; Geerse et al. 2018; Song et al. 2018; Caetano et al. 2019), dementia (Orcioli-Silva et al. 2012; Pieruccini-Faria et al. 2019), stroke (Heeren et al. 2013; Hollands et al. 2015; van Ooijen et al. 2015), and fall experiences (Schoene et al. 2014; Caetano et al. 2018). Compared to young adults (YA), OA and neurological patients were more likely to make stepping errors, took longer to react to stepping targets and obstacles, had significantly greater response variability, and showed a higher risk of falling.

Studies of cognitive-motor tasks which require repeated task switching have demonstrated so-called *proactive* and *retroactive* transfer effects. Proactive transfer refers to the phenomenon that a previously learned cognitive-motor task influences the learning of the subsequent similar task. The retroactive transfer is a reverse phenomenon that subsequently learned cognitive-motor task influences the preservation of the previously learned task (Hanseeuw et al. 2012). Both transfers are known to result in either detrimental (interference) or beneficial (facilitation) effects depending on age, individual cognitive-motor performance level, amount of practice, task difficulty, or task similarity (Hasher et al. 2002; Seidler 2007). The facilitation and interference effects of the proactive and retroactive transfer have been well investigated in terms of cognitive tasks such as recalling different lists of words, in which appropriate executive functions (i.e., working memory, inhibitory control, and cognitive flexibility) are required (Hedden and Park 2001; Hasher et al. 2002; Murphy et al. 2007; Loosli et al. 2014; Redick et al. 2020). Furthermore, previous studies found transfer effects in YA and OA for long-term established human motor skills such as typing, grasping, or assembly tasks (Panzer et al. 2006; Panzer and Shea 2008; Seidler 2007; Sperl et al. 2021b; Verneau et al. 2015), force field adaptations (Brausher-Krug et al. 1996), visuomotor rotations (Krakauer et al. 1999), or static balance (Egger et al. 2021). Those studies also proposed that OA are prone to have more interference effects in the transfer, while YA tend to have more facilitation effects.

It is noteworthy that all the above-mentioned studies refer to simple finger, hand, or foot movements in sitting and static standing positions, so less is known about transfer effects on more complex cognitive-motor skills in old age. Especially, complex motor skills requiring dynamic balance ability using the whole body, such as the VGA, are relevant for studying efficient and independent daily mobility of OA. VGA has been well studied among YA and OA, but there exists no specific test protocol studying the age-related effects of repeated task switching on VGA as well as it remains unclear which type of VGA tasks is appropriate for this aim. Therefore, the present study aimed to examine age-related changes in VGA in a novel VGA test protocol requiring repeated task switching. For this purpose, YA and OA executed two consecutive different VGA tasks which required stepping on or avoiding visual targets while walking on an instrumented treadmill. According to existing evidence regarding the transfer effects between two different tasks, making the movement of a task automatic (i.e., automation or habituation) is the key point to optimize the interferences between both tasks (Sperl and Cañal-Bruland 2019). To overcome such task manipulation, participants should be able to inhibit the automatized or habitual movement to adapt their movement appropriately to the environmental and task change.

To this end, we developed a relatively simple and another relatively complex VGA task based on stepping on and avoiding visual targets while walking, in which participants’ step error and accuracy as well as the interference and learning effects can be assessed. The simple task required participants to follow a repeated simple walking pattern only while stepping on the visual targets, but not while avoiding them. The complex task asked participants to maintain a specific walking pattern, while both stepping on and avoiding the visual targets. We hypothesized that compared to YA, OA overall have more step errors and poorer step accuracy, larger interference effects while switching the tasks, and less learning effects over repeated blocks.

## Methods

### Participants

Fifteen healthy YA (8 females, average age of 27.5 ± 2.9 years) and 16 healthy OA (5 females, average age of 70.9 ± 7.6 years) participated in the study. Recruitment strategies were individual contact and handing out information brochures in diverse facilities such as sports clubs, associations, or communities. Exclusion criteria for both age groups were orthopedic disorders that affect walking on an instrumented treadmill (C-Mill, Motek^®^)’s surface without the support of the handrail, other treatments that could influence the effects of the interventions (e.g., operations in the lower extremity within the last six months), contra-indication to physical activity (e.g., severe osteoporosis, heart failure), severe uncorrected visual deficits, or moderate to severe cognitive impairments. All participants provided written informed consent before testing began. All aspects of the study conformed to the principles described in the Declaration of Helsinki. This study was approved by the Ethics Committee of the German Sport University Cologne (Nr 077/2022).

### Experimental materials and procedures

Participants’ VGA was assessed based on step errors and step accuracy on the C-Mill. C-Mill enables experiments with various augmented and pre-programmed visual stimuli on the surface, such as goal-directed stepping, obstacle avoidance, or speed adjustment. A force plate embedded under the C-Mill’s surface provided kinetic data (center of pressure, CoP) for dynamic stepping abilities in each task (sampling rate: 500 Hz). For the current study, we developed a specific test protocol based on the goal-directed dynamic stepping task of the C-Mill. This test protocol was fine-tuned in a preparation phase (8 YA and 2 OA).

**Fig. 1:**
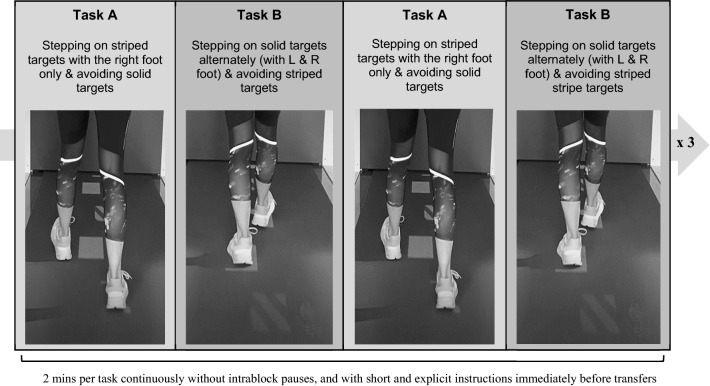
Overview of one block of VGAt on the C-Mill. A–B–A–B study design was applied. Participants repeated the block three times with breaks between blocks, but not between tasks. In Task A, participants stepped on striped targets with their right foot only while avoiding solid targets by stepping anywhere on the surface

Our voluntary gait adaptability task (VGAt) comprised two different principles i.e., stepping on or avoiding colored visual targets (rectangle white solid and red/white striped targets; from this onward named as ‘solid’ and ‘striped’ targets) on the walking surface (see Fig. 1). All participants wore a safety harness attached to the top rail of the C-Mill to avoid possible falls while walking. They first practiced walking on the C-Mill at their comfortable belt speed for approximately 1–2 min to be familiarized (depending on their experience with walking on a treadmill). Subsequently, participants were required to perform two specific C-Mill tasks (slalom and track) to measure their individually comfortable belt speed, because walking speed is known to decrease when additional visual or auditory stimuli are given (Peper et al. 2015). For better understanding, participants were explicitly instructed by the experimenter using photos on a smart tablet to make sure which visual stimuli they will have (i.e., for slalom: a s-shaped path; for track: green or red/white striped targets). The purpose of the photo-based instruction was to instruct the participants more clearly, as they were completely unfamiliar with the visually guided walking on the C-Mill, so the verbal instruction was not enough for them to understand the tasks, which we noticed in the pilot study. Both tasks were suitable as a kind of warm-up task, since both tasks differed from the main stepping target task, but the participants could understand the basic principle of visually guided walking on the C-Mill. During both tasks, the individual’s belt speeds on the C-Mill were defined as in previous studies using the C-Mill (Timmermans et al. 2019): slowly increasing the belt speed in steps of 0.1–0.5 km/h until the participants report it as comfortable. Those two indications of comfortable belt speed were averaged and taken to represent the individual’s belt speed for the VGAt. After measuring individual belt speed, individual step length was measured based on that individual belt speed while walking on the C-Mill without any visual stimuli for approximately 30 s.

**Fig. 2:**
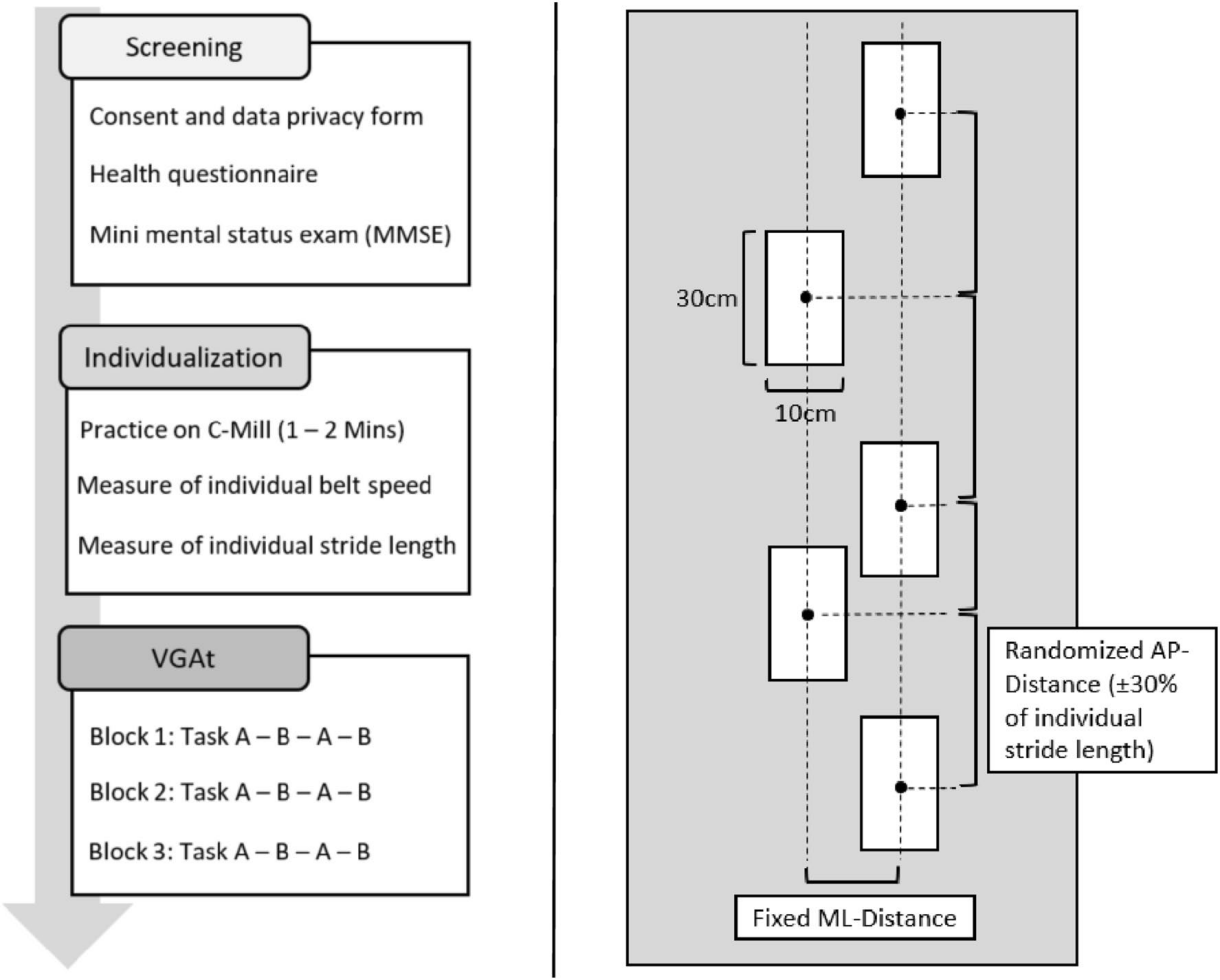
The workflow of the experiment (left) and the overview of visual targets (right)

Our VGAt consisted of two different gait adaptability tasks (Task A and B). Participants carried out both tasks twice in a block in a sequence order alternately (A–B–A–B, 2 min per task) without any intrablock breaks (Block 1, 8 min in total). They repeated the block three times (1 min break between blocks). After the preparation, participants were explicitly instructed by the experimenter using an instruction video on the smart tablet. The experimenter showed the video of each task as well as an overview of the whole procedure to ensure that the participants understood the tasks. Participants were also asked to describe the task and procedure. To avoid transfer effects due to misunderstanding the tasks, participants were given explicit instructions on the whole procedure. A sequence of stepping targets (length: 30 cm, width: 10 cm; solid and striped targets), which were colored either in plain white without any patterns or in a red and white diagonal stripe pattern was projected onto the C-Mill’s walking surface. Distances between stepping targets were randomized in the anterior–posterior (AP) direction (± 30% of individual stride length) but fixed in the mediolateral (ML) direction (see Fig. 2). The appearing proportion of solid targets was 60% and of striped targets was 40%.

In Task A, participants were asked to avoid solid targets but step on striped targets with their right foot only. When avoiding the striped targets, participants could step anywhere on the C-Mill’s surface (i.e., a greater number of steps than the number of solid targets is available, but the same number of steps of the right foot as the number of striped targets). Since we tried in this task to make participants’ walking automatically to optimize the interference effects while the change of task, Task A was set up with a relatively compact principle (notice only the right foot) compared to Task B. To switch from Task A to Task B or vice versa, participants were verbally instructed 15 s before the end of 2 min of each task. In Task B, participants were required to step on the solid targets with their left and right foot alternately (distinguishable left and right sides) while avoiding striped targets. When avoiding striped targets, participants may have stepped on only the outside of the striped targets but not anywhere on the walking surface (i.e., the same number of steps as the striped and solid targets). Since both tasks encompassed the same visual stimuli (solid or striped visual targets) but required different motor behaviors (walking pattern), different VGA was expected depending on the factor Age (young and old) and Trial (six trials for Task A and B respectively), as well as their interaction effects.

### Data registration and analysis

Outcome measures of VGAt were percentage of step errors recorded by a camera and the step accuracy recorded by the force plate embedded in the C-Mill’s walking surface. We first analyzed step errors from recorded videos based on the following criteria:

- *step errors in Task A* (Demand: Stepping on striped targets only with the right foot and avoiding solid targets without a certain step pattern)
  o when stepping on solid targets with the left or right foot
  p when stepping on striped targets with the left foot
- *step errors in Task B* (Demand: Stepping on the left and right solid targets with left and right foot alternately and avoiding striped targets with a certain step pattern)
  o when stepping on striped targets with the left or right foot
  p when stepping on left and right solid targets with false foot
  q further steps on the walking surface more than the certain step pattern

Step accuracy was calculated using the means of the CoP difference values between participants’ foot center position and stepping stones’ center position data in both AP and ML directions. Participants’ gait characteristics for the VGAt (step length left and right, belt speed, and the total number of steps) were first compared between both age groups by independent* t*-tests (see Table 1).

**Table 1:** Descriptive statistics

Characteristics	YA, *n* = 15	OA, *n* = 15
Age (mean ± SD)	27.5 ± 2.9	70.9 ± 7.6
Gender distribution (% women)	53.3	31.3
Height (m)	1.72 ± 0.07	1.71 ± 0.10
Weight (kg)	66.7 ± 11.8	75.3 ± 14.0
Step length left (m)	0.63 ± 0.06	0.51 ± 0.08
Step length right (m)	0.63 ± 0.06	0.51 ± 0.09
Total number of steps in Task A*	97.19 ± 10.21	102.59 ± 11.94
Total number of steps in Task B**	192.53 ± 17.51	191.98 ± 22.96
Belt speed (km/h)	3.71 ± 0.46	3.03 ± 0.51

*Number of steps of right foot only

**Number of steps of left and right foot

**Fig. 3:**
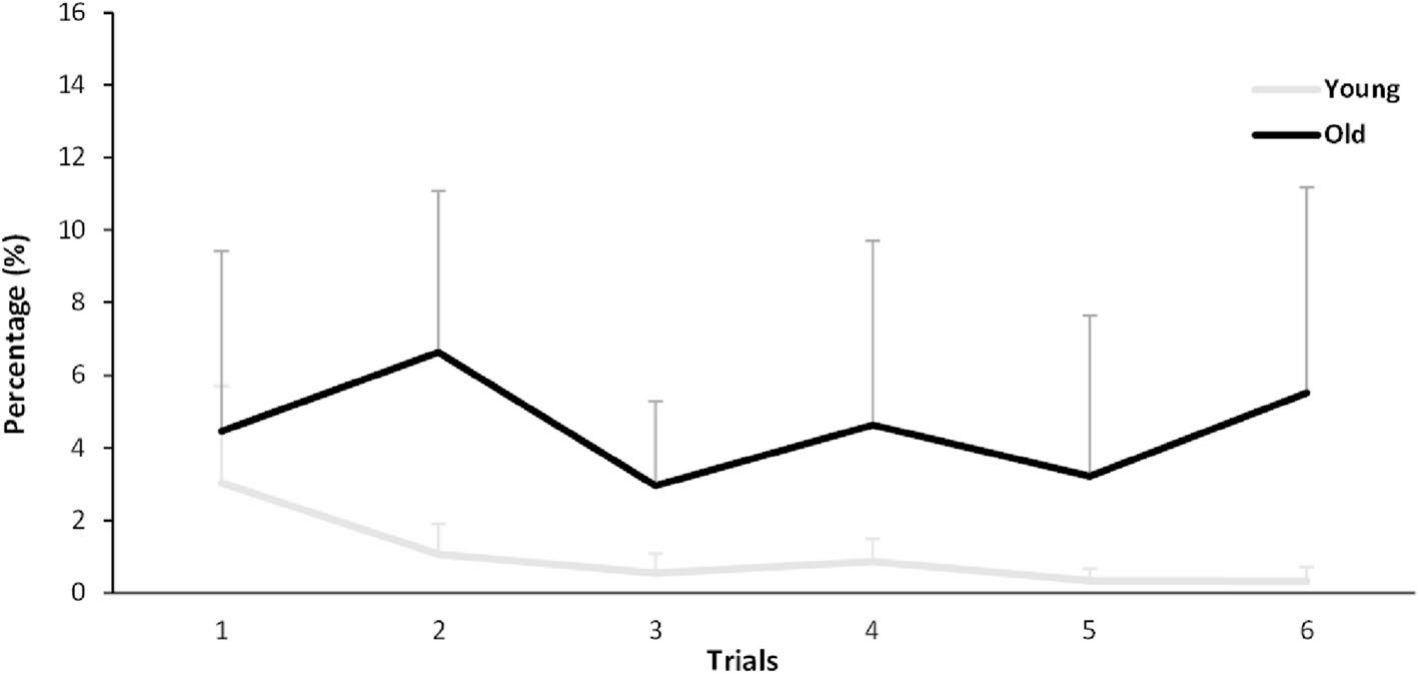
Step errors in Task A

**Fig. 4:**
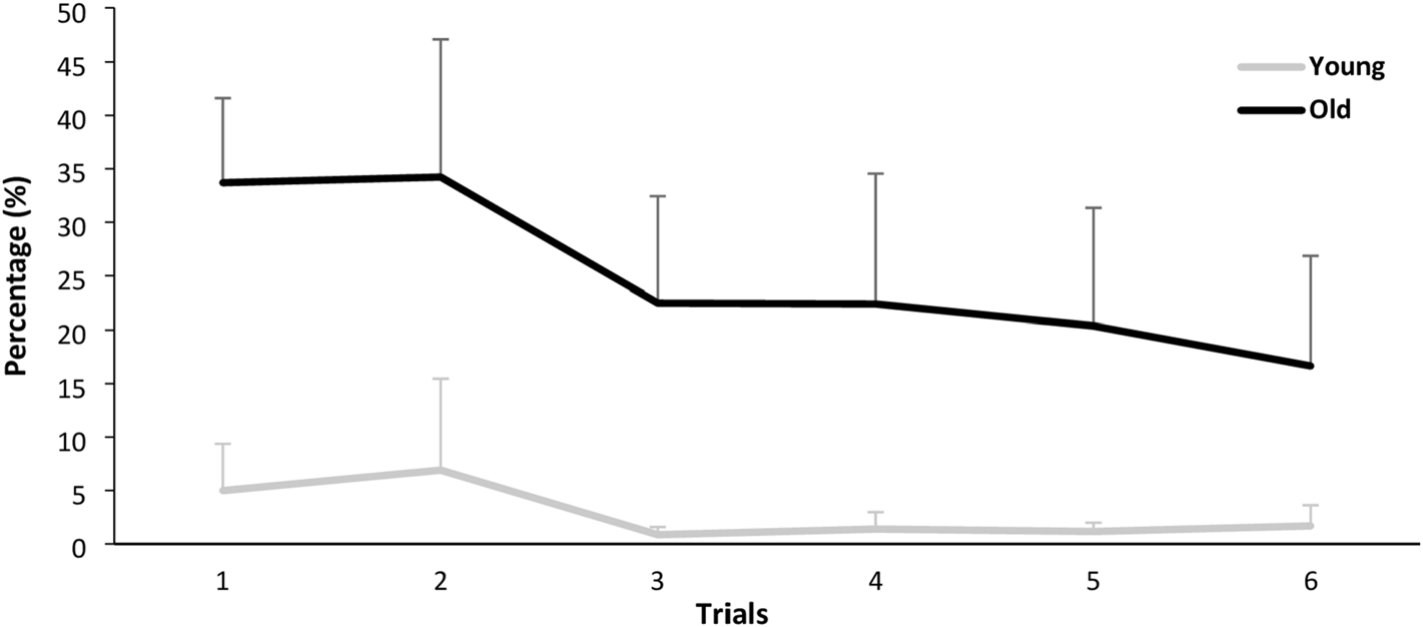
Step errors in Task B

**Fig. 5:**
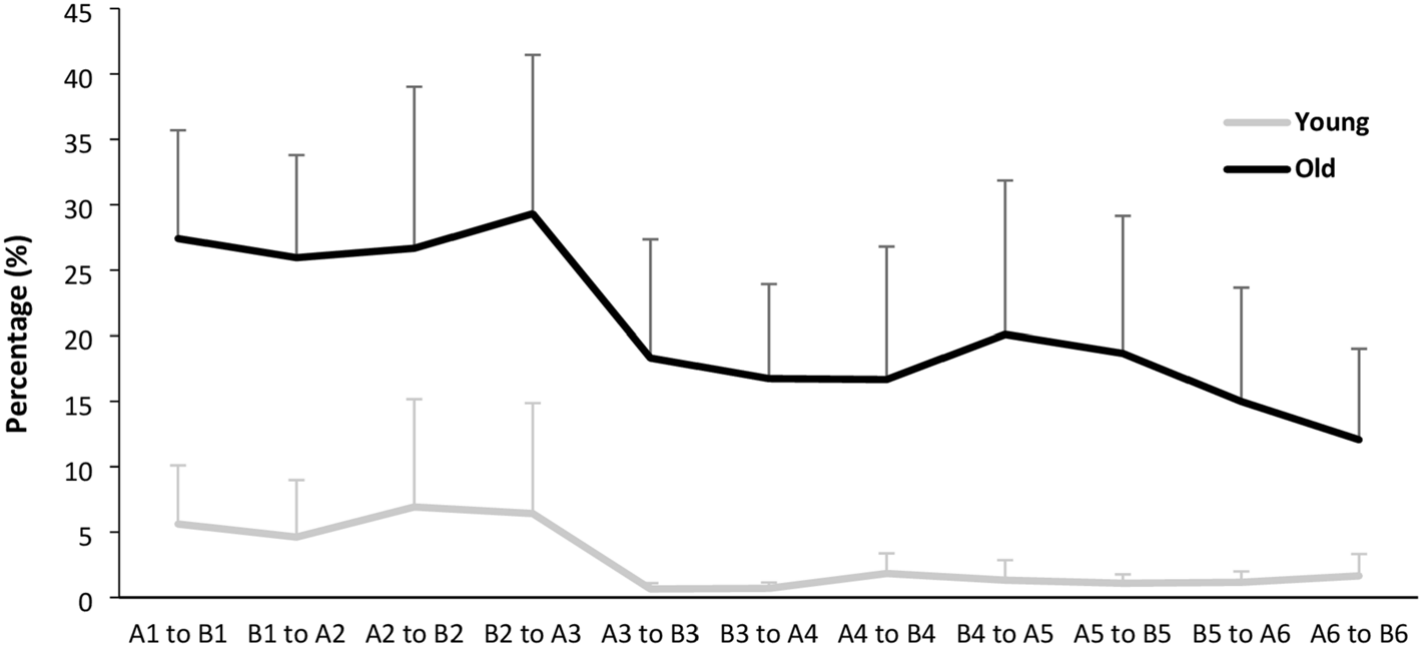
Age-related degree of interference effects between Task A and B

We calculated the number of step errors in percent. After the confirmation of normal distributions (Kolmogorov Smirnov test, *p* > 0.05), the percentage of step errors between both age groups was calculated by the analysis of variance with repeated measures (ANOVA) with the factor Age (young-old) and Trial (A_1_,…, A_6_; B_1_,…, B_6_), separately in Task A and B (see Figs. 3 and 4). As a second step, we calculated the difference value of the step errors between each trial over three blocks independently of Task A or B for interference effects. After the confirmation of normal distributions, the interference effects between each trial over three blocks were calculated by the analysis of variance with repeated measures (ANOVA) (see Fig. 5). In the step accuracy, we calculated the analysis of variance with repeated measures (ANOVA) with the factor Age (young-old) and Trial (A_1_,…, A_6_; B_1_,…, B_6_), respectively, in AP and ML direction.

## Results

### Participants’ characteristics

One male OA (AP_EG1_05) was excluded from data analysis, because he was not able to follow our instructions in the main task: his data were therefore not commensurate to be interpreted. Table 1 shows descriptive statistics of all participants. There were significant group differences in step length of left (*t*(28) = 4.969; *p* < 0.001) and right (*t*(28) = 4.543; *p* < 0.001) foot, and individual belt speed (*t*(28) = 3.836; *p* < 0.001), but not in the total number of steps in Task A (*t*(28) = − 1.513; *p* = 0.142) and B (*t*(28) = 0.090; *p* = 0.929).

### Step errors

Figure 3 shows the participants’ learning performance in Task A over three blocks (a total of six trials). The analysis of variance (ANOVA) with repeated measures yielded significance of Age (*F*(1, 84) = 12.791; *p* < 0.001; *ŋ*^2^ = 0.132), but no significance of Trial (*F*(5, 84) = 0.538; *p* = 0.747; *ŋ*^2^ = 0.031) and Age*Trial (*F*(5, 84) = 0.445; *p* = 0.816; *ŋ*^2^ = 0.026).

Figure 4 shows the participants’ learning performance in Task B over three blocks (a total of six trials). The analysis of variance (ANOVA) with repeated measures yielded significance of Age (*F*(1, 84) = 84.861; *p* < 0.001; *ŋ*^2^ = 0.503) and of Trial (*F*(5, 84) = 2.535; *p* = 0.035; *ŋ*^2^ = 0.131), but no significance of Age*Trial (*F*(5, 84) = 0.758; *p* = 0.583; *ŋ*^2^ = 0.043).

Figure 5 illustrates the interference level between both Tasks A and B. The analysis of variance (ANOVA) with repeated measures yielded significance of Age (*F*(1, 154) = 145.880; *p* < 0.001; *ŋ*^2^ = 0.486) and of Trial (*F*(10, 154) = 2.279; *p* = 0.016; *ŋ*^2^ = 0.129), but no significance of Age*Trial (*F*(10, 154) = 0.620; *p* = 0.795; *ŋ*^2^ = 0.039).

### Step accuracy

Table 2 shows the means of step accuracy in both AP and ML directions separately. In Task A, age-related differences were shown in the AP direction (*F*(1, 75) = 12.147; *p* < 0.001; *ŋ*^2^ = 0.139) but not in the ML direction (*F*(1, 75) = 0.113; *p* = 0.737; *ŋ*^2^ = 0.002). Trial-related differences were not shown in AP (*F*(5, 75) = 1.667; *p* = 0.153; *ŋ*^2^ = 0.100) and ML direction (*F*(5, 75) = 0.138; *p* = 0.983; *ŋ*^2^ = 0.009). The interaction effects between Age*Trial were not significant both in AP (*F*(5, 75) = 0.246; *p* = 0.941; *ŋ*^2^ = 0.016) and ML direction:* F*(5, 75) = 1.234; *p* = 0.302; *ŋ*^2^ = 0.076).

**Table 2:** Means and standard deviations of step accuracy in AP and ML direction

Task A	Anterior–posterior (AP)		Mediolateral (ML)	
	YA (cm, mean ± SD)	OA	YA	OA
A_1__Block 1	**0.128** ± 0.063	0.170 ± 0.097	0.060 ± 0.024	**0.040** ± 0.027
A_2__Block 1	**0.089** ± 0.079	0.150 ± 0.084	0.047 ± 0.031	**0.045** ± 0.023
A_3__Block 2	**0.095** ± 0.058	0.155 ± 0.115	0.052 ± 0.031	**0.051** ± 0.035
A_4__Block 2	**0.067** ± 0.051	0.106 ± 0.092	0.054 ± 0.036	**0.047** ± 0.023
A_5__Block 3	**0.090** ± 0.081	0.143 ± 0.096	**0.046** ± 0.019	0.050 ± 0.033
A_6__Block 3	**0.111** ± 0.096	0.120 ± 0.040	**0.042** ± 0.024	0.052 ± 0.039

Task B	YA	OA	YA	OA
B_1__Block 1	0.110 ± 0.041	**0.091** ± 0.043	**0.041** ± 0.024	0.065 ± 0.058
B_2__Block 1	0.101 ± 0.050	**0.096** ± 0.045	0.067 ± 0.048	**0.040** ± 0.039
B_3__Block 2	0.105 ± 0.067	**0.086** ± 0.042	0.059 ± 0.037	**0.054** ± 0.045
B_4__Block 2	0.106 ± 0.033	**0.073** ± 0.041	0.050 ± 0.058	**0.038** ± 0.032
B_5__Block 3	0.100 ± 0.038	**0.088** ± 0.043	0.064 ± 0.045	**0.056** ± 0.058
B_6__Block 3	0.104 ± 0.028	**0.084** ± 0.052	0.095 ± 0.071	**0.055** ± 0.040

*Better step accuracy values are in bold

In Task B, age-related differences were shown in AP (*F*(1, 74) = 7.893; *p* = 0.006; *ŋ*^2^ = 0.096), but not in ML direction (*F*(1, 74) = 1.882; *p* = 0.174; *ŋ*^2^ = 0.025). Trial-related differences were not shown in AP (*F*(5, 74) = 0.224; *p* = 0.951; *ŋ*^2^ = 0.015) and ML direction (*F*(5, 74) = 1.540; *p* = 0.188; *ŋ*^2^ = 0.094). The interaction effects between Age*Trial were not significant both in AP (*F*(5, 74) = 0.299; *p* = 0.912; *ŋ*^2^ = 0.020) and in ML direction *(F*(5, 74) = 1.882; *p* = 0.323; *ŋ*^2^ = 0.074).

## Discussion

The present study aimed to examine age-related changes in VGA in a novel VGA test protocol requiring repeated task switching. We hypothesized that compared to YA, OA overall have more step errors and poorer step accuracy, larger interference effects, and less learning effects over repeated blocks. Performances were determined by the percentage of step errors and step accuracy on two different stepping tasks (Task A and B) that the participants performed alternately without any breaks within a block (Task A–B–A–B), but with breaks between blocks (Block 1–2–3). Our results showed that OA overall made significantly more step errors both in Task A and B than YA. They also showed more interference effects over all trials than YA. Participants’ step accuracy differed significantly in the AP direction both in Task A and B, but not in the ML direction.

### Step errors and interference effects

Participants’ performances in step errors were analyzed over three subsequent blocks in Task A and B respectively. OA showed significant performance development in Task B but not in Task A. However, in Task A, OA generally tended to show a slight performance reduction after learning Task B, but a slight improvement after the block breaks (see Fig. 3). This may be explained by the retroactive transfer effects that subsequently learned motor skill influences the previously learned skill, which may attribute to OA’s impaired working memory and the ability to adapt to complex and dynamic tasks. Indeed, there is already known evidence that working memory capacity is an important factor in resistance to proactive interference (May et al. 1999). Our Task A was influenced by Task B, especially in OA, but the significance was not shown. On the other hand, OA did not show any significant performance development. The absence of performance development of YA may be due to the ceiling effect. However, we preliminarily modified our test protocol through several tests in an internal pilot phase and have found that overly complex tasks may not be appropriate to induce interferences or facilitations in YA and OA. In this sense, our VGAt test protocol might have been a simple motor skill task for YA, but we could see that aging affects a specific basic human gait skill (i.e., gait adaptability) necessary for safe mobility in old age.

More importantly, the performance development of OA in Task B would support the existing evidence that OA can achieve considerable increases in motor performance and that their learning capability is preserved (Voelcker-Rehage 2008). Since the extent of learning capability varies with the type of motor skills (fine or gross motor skill) or the task complexity, the performance development of our OA would provide us the first evidence for the learning capability in voluntary gait adaptability in old age. Unlike YA, however, OA did not reach optimal levels on both Tasks A and B. If OA had performed more than three blocks, they could have reached the optimal level, but considering problems such as reduced attention due to too long task time, additional blocks may have had another effect on the results. Indeed, two OA reported that three blocks (2 min per task and 8 min per block) were too hard and long to keep their attention for the task. Therefore, studying when OA can reach the optimal level while maintaining attention also seems necessary in further studies.

The interference effects were analyzed based on the step errors. Our results were in line with previous studies that demonstrated more interference effects in OA than in YA (Hasher et al. 2002; Seidler 2007). Most of all, the interference effects are related to short-term memory which is crucial for normal cognitive and motor processing. In aging studies, it has been repeatedly observed that OA are prone to have more difficulty resolving interference effects in short-term memory (Manard et al. 2014). Inhibitory control is also assumed to play an essential role in the resolution of interference effects (Friedman and Miyake 2004). The increase of interference effects in OA may result from a less efficient inhibitory control process than YA. According to existing findings and as we described in the theoretical background, the amount of interference effects is related to baseline performance and the degree of movement automaticity (Sperl et al. 2021a). That is why our Task A was relatively simple and required a simple movement principle i.e., stepping on striped visual targets only with the right foot. We assumed that the movement in Task A was automated during the test time (2 min) and therefore the ability to suppress these automated visual perceptions and foot placement would be impaired during task switching.

### Step accuracy

In step errors, we found significant age-related differences in the AP direction but not in the ML direction. One possible reason would be the randomized distances between visual targets in AP direction and the fixed distance in ML direction. As described in Fig. 2, distances between visual targets were randomized in the AP direction (± 30% of individual stride length) but fixed in the ML direction. It was determined in our pilot phase that randomized distances in ML direction make our VGAt too difficult both for YA and OA, especially in order to keep their balance, step pattern, and step rhythm after avoiding visual targets. Therefore, we set the distance in the ML direction to 10 cm, which most participants in the pilot phase found most comfortable. This might have contributed to the absence of age-related differences in the ML direction since OA were able to keep their dynamic balance while walking as YA did. Such interpretation can also be related to the interesting results that OA showed worse step accuracy in Task A (easier task), but even better in Task B (more complex task). According to previous studies regarding obstacle avoidance, OA generally tend to decrease their step length, velocity, or landing distance and increase their trunk range of motion in the roll, pitch, and yaw directions (Weerdesteyn et al. 2005; Lowrey et al. 2007). OA may have chosen a more cautious strategy to ensure safety and carefully execute our VGAt, while YA may have focused more on not making step mistakes than on stepping accurately. To ensure this interpretation, further studies appear necessary to examine what/where OA and YA pay attention in our VGAt (e.g., eye tracking study or development of a specific test protocol about the focus of participants).

### Remaining challenges

Although the current study has the strength of examining the effects of repeated task switching on VGA of healthy OA, other important factors potentially have a large influence on their VGA, such as different task complexity of Task A and B, individually different belt speed, and randomized AP distances between visual targets. Regarding the task complexity, it is unclear whether Task A was too easy and Task B rather too difficult for OA, i.e., whether the step errors in both tasks were due to the tasks per se or the task-switching effect as interpreted. However, the learning curves on Task A indicate that OA showed a slight increase in errors after learning Task B, while YA did not show such an increase (see Fig. 3). This means our test protocol was adequate to examine the effects of repeated task switching on VGA. Further research should consider increasing the complexity of Task A to the same level as that of Task B to see whether the effect of repeated task switching is more pronounced. The inference about which specific aspect is responsible for slowing voluntary movement and motor processing in old age should be considered in future research.

We deliberately chose to use individualized conditions for belt speed and step length, in order to test VGA of OA and YA under conditions that were based on their preferred walking patterns rather than a standard belt speed. Using the same belt speed for all participants would possibly affect outcomes by creating conditions that are experienced differently by YA and OA (e.g., 3.0 km/h would have been appropriate for OA but too slow for YA, and vice versa for 4.5 km/h). The randomized anterior–posterior distances between visual targets (± 30% of individual step length) also differed between participants. Ultimately, the individualized conditions that were applied in our protocol created conditions that were mechanically less challenging for OA (i.e., lower speed smaller distances for step targets). Nevertheless, our results do show clear age-related differences in the VGA-related tasks.

## Conclusion

Taken together, our results suggest that OA appear more interfered by repeated task changes both in step errors and accuracy than YA, which is in line with existing evidence from cognitive functions and simple motor tasks. Our results provide new evidence of age-related interference effects while repeated task switching in motor learning and performance based on dynamic balance control while walking. Since the significant main effect of trial for Task B, but not Task A appears to be due to different task complexity, further studies are needed to determine the effect of task complexity or task switch timing on participants’ VGA in motor skill change in more depth.

## Data Availability

The datasets generated during and/or analyzed during the current study are available from the corresponding author on reasonable request.
